# Intratympanic Steroid Treatment for Idiopathic Sudden Sensorineural Hearing Loss after Failure of Intravenous Therapy

**DOI:** 10.5402/2012/647271

**Published:** 2012-02-19

**Authors:** Emanuele Ferri, Antonio Frisina, Anna Chiara Fasson, Enrico Armato, Giacomo Spinato, Maurizio Amadori

**Affiliations:** ^1^Otologic Surgery Unit, Otorhinolaryngology Department ULSS 13, General Hospitals of Dolo and Mirano, Via Mariutto 76, 30035 Mirano, Italy; ^2^Otorhinolaryngology Department, San Bortolo Hospital, Via F. Rodolfi, 37, 36100 Vicenza, Italy; ^3^ENT Clinic, Head and Neck Department, University of Trieste, Hospital of Cattinara, Strada di Fiume 447, 34149 Trieste, Italy

## Abstract

*Purpose*. The aim of this study is the investigation of the effectiveness of intratympanic steroids therapy (IST) in patients with idiopathic sudden sensorineural hearing loss (ISSHL) who had not responded to intravenous treatment, evaluating the overall hearing recovery and comparing the results with different variables. *Materials and Methods*. Our study consisted of 55 patients with refractory ISSHL who, at the end of 10 days of therapy with intravenous steroids, had puretone 4-frequency average (PTA) of worse than 30 dB. The patients received 0.5 mL of methylprednisolone by direct intratympanic injection. The procedure was carried out up to 7 times within a 20-days period. Statistical analysis was carried out. Results. Overall 29 patients (52.7%) showed improvement in PTA, 24 (43.8%) had no change in hearing, and 2 (3.5%) worsened. There was a significant statistical correlation between hearing recovery and time to onset of symptoms, severity of hearing loss and frequency of hearing loss. *Conclusions*. IST is an effective and safe therapy in sudden sensorineural hearing loss cases that are refractory to standard treatment. The earlier IST, the hearing losses less than 90 dB and the involvement of the low frequencies seem to influence positively the hearing recovery.

## 1. Introduction

Nearly 60 years after the first report of idiopathic sudden sensorineural hearing loss (ISSHL) [1], the otologists are still searching answers to the etiology, physiopathology and therapeutical management of this disorder. The most common theories of the etiology of ISSHL include viral infection, vascular occlusion with microcirculatory disturbances, immunologic diseases, and intralabyrinthine membrane breaks [2–6]. ISSHL usually presents as an acute unilateral deafness of more than 30 dB hearing loss involving three contiguous frequencies, with an abrupt onset, generally within three days or less. It occurs in 5–20 cases per 100.000 population. This is approximately the same incidence as Ménière's syndrome (15 per 100000) and twenty times more common than acoustic neurinoma (1 per 100000) [2, 7]. The true incidence of ISSHL is probably underestimated because many who recover hearing early are unlikely to seek medical therapy. 

Many treatments for ISSHL have been tested and found ineffective. These include hyperbaric oxygen, agents that decrease blood viscosity (osmotic diuretics, pentoxifylline, procaine, and heparin), vasodilator drugs (histamine, papaverine, verapamil, and carbogen), free radical scavenging vitamins, gingko biloba, and magnesium. At this time, the only treatment for ISSHL shown effective in controlled clinical trials is systemic corticosteroid therapy with high dose of prednisone taper. The reported success rate is around 50 to 80%, whereas the spontaneous recovery rate is approximately 30 to 60% [8–10]. Despite high reported spontaneous recovery rates, the practical experience of many otologists suggest that, hearing recovery is poor in those patients who have failed systemic intravenous therapy [11–13].

Although the safety and efficacy of intratympanic steroids therapy (IST) have not been studied in a randomized clinical trial, there are many evidences to suggest that IST improves treatment success by increasing intracochlear corticosteroid and reducing the incidence of toxic side effects. The optimal drug, the dosage, the treatment schedule, the duration of treatment, and the standard protocol universally accepted are not known yet.

The aim of this study is the investigation of the safety and effectiveness of IST in the treatment of ISSHL after failure of intravenous steroid therapy, with special attention for the correlations between hearing recovery and time to onset of therapy, severity and frequencial range of hearing loss, age of the patient, and status of the contralateral ear.

## 2. Materials and Methods

Between January 2005 and December 2008, a nonrandomized prospective clinical trial was conducted on 158 patients with a diagnosis of ISSHL. The study was approved by the local Institutional Review Board, and each patient provided informed consent. The patients were hospitalized at the Otosurgery and Audiology Units of General Hospital of Dolo (Venice) and San Bortolo Hospital of Vicenza. Physical examination, pure-tone and speech response audiometry, tympanometry, syphilis serology, autoimmune antibody tests, auditory brainstem response (ABR), and temporal bone high-resolution computed tomography (CT) were performed. Cerebral magnetic resonance imaging (MRI) was performed only if a retrocochlear lesion was suspected by means of ABR and high-resolution CT.

All patients were treated intravenously with 4–8 mg of intravenous betamethasone for 10 days.

Auditory function was determined by pure-tone audiometry; the mean hearing levels were expressed as the average of hearing thresholds at 0.5, 1, 2, and 3 kHz (4-tone average) (PTA), according to the guidelines of the Committee on Hearing and Equilibrium of the American Academy of Otolaryngology-Head and Neck Surgery. Auditory measurements were performed before, during, and 3-month after treatment, according to Siegel's criteria for hearing improvement [14]. “Complete recovery” was defined as more than 30 dB hearing gain and as final hearing better than 25 dB, “partial recovery” as more than 15 dB hearing gain and as final hearing between 25 and 45 dB, “slight improvement” as more than 15 dB hearing gain but with a final hearing poorer than 45 dB, and “no improvement” as less than 15 dB hearing gain and final hearing poorer than 75 dB.

After intravenous steroid therapy, if the patients showed a recovery less than 50%, they were enrolled for IST as salvage treatment.

### 2.1. Inclusion Criteria

Patients with ISSHL were enrolled and treated with IST if they:

- had a sudden unilateral hearing loss of at least 30 dB across 3 contiguous frequencies occurring in less than 72 hours or progressively over several days, but with an onset within 30 days;
- had begun steroid treatment (4–8 mg of intravenous betamethasone for 10 days) within 10 days of hearing loss onset;
- recovered less than 50% of their preloss hearing during steroid treatment and presented for IST within 1 month of onset;
- had no retrocochlear pathology as demonstrated by negative MRI scan;
- were age 18 years or older.

### 2.2. Exclusion Criteria

Patients with ISSHL were excluded when they presented hearing loss history with onset over 30 days, oncologic history with recent chemotherapy or radiation therapy, autoimmune diseases, congenital cochlear malformations, Ménière's disease, acute or subacute otitis media with abnormal tympanometry, neurological disorders, recent use of ototoxic medications, severe liver or renal dysfunction, pregnancy, recent trauma.

### 2.3. Operative Procedure of Intratympanic Injection

The operative procedure of intratympanic steroid injection was performed under a microscope and with patient in supine position. After the surgeon confirmed intact tympanic membrane and middle ear status, local anaesthesia was administered with a cotton ball soaked with lidocaine 10% pump spray (Xylocaine), which was applied on the tympanic membrane for 20 minutes. While the patient tilted the head 45° to the healthy side, a 25-gauge spinal needle was introduced into the posteroinferior portion of membrane and 0.4-0.5 mL of methylprednisolone (40 mg/mL) was instilled through this site. The patient was instructed to avoid swallowing or moving for 30 minutes, remaining in the same position. IST was performed on the 1st, 3rd, 5th day up to 7 total injections, one every two or three days.

### 2.4. Statistical Analysis

Statistical analysis was carried out using the Student's *t*-test for continuous variables and Fisher's test for categorical variables. A *P* value of less than  .05 was considered statistically significant.

## 3. Results

After inclusion and exclusion criteria were applied, 55 patients were available for the study.

There were 24 men (43.6%) and 31 women (56.4%). The mean age at enrolment for all patients was 49.7 years and ranged from 18 to 83 years. The mean age for the men was 53.2 years and for the women was 46.1 years.

### 3.1. Overall Hearing Recovery

**Figure 1:**
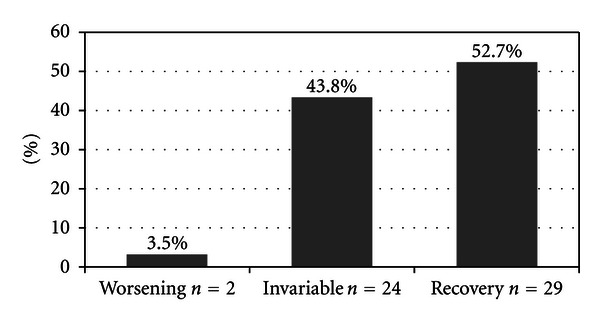
Overall hearing recovery.

**Figure 2:**
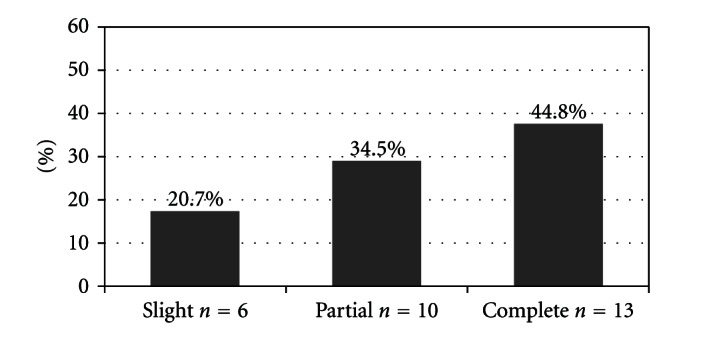
Hearing recovery according to Siegel's criteria.

Overall, 29 patients (52.7%) showed improvement in PTA, 24 (43.8%) had no change in hearing, and 2 (3.5%) worsened (Figure 1). According to Siegel's criteria, 13 patients showed “complete recovery” with a mean gain of 36.2% (range 12.4% to 86.9%); 10 patients showed “partial recovery” with an average improvement of 18.9% (range 7.8% to 69.2%); 6 patients had a “slight recovery” with a mean gain of 16.2% (range 6.1% to 49.8%) (Figure 2).

### 3.2. Recovery Related to Time to Onset of Symptoms

The average number of days from onset of symptoms to IST was 33 days with a range of 5 days to 96 days. For the group that responded to IST with a “complete recovery” (*n* = 7), the median was 12 days; for the group that responded to IST with a “partial or slight recovery” (*n* = 22), the median was 23 days; for the group that did not respond (*n* = 26), the mean was 34 days. Statistical analysis shows that there is a significant correlation between hearing recovery and time to onset of symptoms; patients that started IST soon after failures of systemic therapy was detected had an evident advantage (*P* = .007 Fisher's test).

### 3.3. Recovery Related to Severity of Hearing Loss

**Figure 3:**
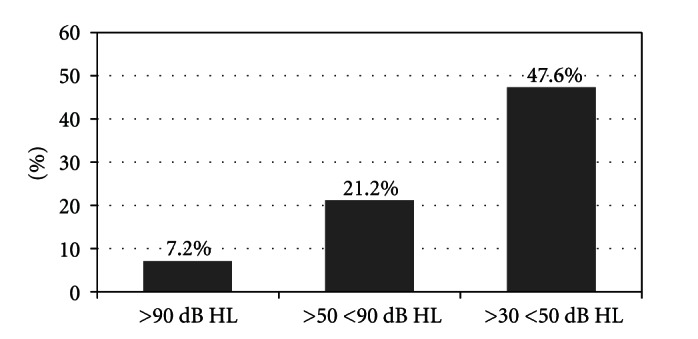
Recovery rate related to severity of initial hearing loss.

A total of 16 patients (29.1%) had hearing loss greater than 90 dB with an improvement rate of 7.2%; a total of 29 patients (52.7%) had hearing loss of 90 dB or less and greater than to 50 dB with improvement rate of 21.2%; a total of 10 patients (18.2%) had hearing loss less than 50 dB and greater than 30 dB with an improvement rate of 47.6% (Figure 3). Patients with severe losses greater than 90 dB had a poorer recovery (7.2%) compared with losses less than 90 dB (35.6%) (*P* = 0.06 Fisher's test).

### 3.4. Recovery Related to Age of the Patient

Hearing recovery related to patient's age was studied. Fifty-seven percent of patients were under 60 years of age and had an overall recovery rate of 26%. Forty-three percent of patients were 60 years of age or older and had an overall recovery of 32%. Statistical analysis shows no significant correlations between age and improvement after IST (*P* = .08 Fisher's test).

### 3.5. Recovery Related to Status of the Controlateral Ear

A total of 76.5% of patients had normal hearing in the contralateral ear. The recovery rate in this group was 31.5%. Only 23.5% of patients had abnormal hearing in the opposite ear. The recovery rate in this group was 22.5%. Statistical analysis shows no significant correlations between recovery and situation of the contralateral ear (*P* = 1.2 Fisher's test).

### 3.6. Recovery Related to Frequency of Hearing Loss

**Figure 4:**
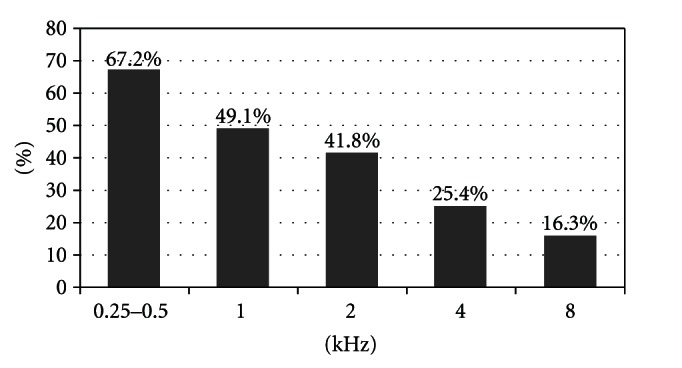
Recovery rate related to frequency of hearing loss.

We have analyzed the hearing recovery for each frequency (0.25, 0.5, 1, 2, 4, and 8 kHz) of hearing threshold. A total of 37 patients (67.2%) showed improvement over 30 dB on hearing gain for the frequency of 0.25 and 0.5 kHz. The same result was obtained with 1 kHz frequency in 27 patients (49.1%), with 2 kHz frequency in 23 patients (41.8%), with 4 kHz frequency in 14 patients (25.4%), and with 8 kHz frequency in 9 patients (16.3%) (Figure 4). Statistical analysis shows a significant correlation between recovery and low frequencies (0.25 and 0.5 kHz) of hearing threshold (*P* = .06 Fisher's test).

## 4. Discussion

The ISSHL is a very frightening and incapacitating event, and it severely impairs patient's life quality and social interaction. Considering the high rate of spontaneous recovery, it is difficult to determine if any therapeutic intervention actually improves the hearing. The natural history of untreated patients with ISSHL states that the recovery rates varies from 31% to 65% [7, 8, 13, 15], while the hearing recovery in treated patients ranges from 35% to 89% [12, 13]. Such a result may be related to different factors: the variable treatment protocols, the type of steroid used, the length of therapy, the patient data, the severity of hearing loss, the duration from onset of symptoms to treatment, the method of statistical analysis. At this time, steroids systematic administration is considered to be the most commonly accepted treatment for ISSHL.

In 2002 Gloddek et al. demonstrated the immunologically mediated vasculitis relation with ISSHL pathogenesis. The role of endothelial cells in this mechanism is inferred, and these cells are thought to promote vasculitis by secreting cytokines [16]. Moreover, ISSHL seems be considered the result of abnormal activation of endocochlear nuclear factor-*κ*B. This is a molecular transcription factor that plays a key role in the normal cellular physiology and in mediating the cellular responses to a pathogenic stress (infectious, mechanical, or osmotic), with stimulation of synthesis of cytokines and alterations of homeostatic balance of the inner ear. The transient activation of this system might be related to a spontaneous recovery, whereas a prolonged stimulation should lead to an irreversible damage of cochlear cells (in most cases, the atrophy of Corti's organ) [17].

The precise mechanism through which steroids may improve hearing remains unknown; both glucocorticoid and mineralcorticoid receptors may be found in the inner ear [18]. The main roles of steroids in the treatment of ISSHL are: (i) the protection of cochlea from the harmful effects of inflammatory mediators, such as the tumor necrosis factor (TNF-*α* and NF-*κ*B) and cytokines (interleukin 1 and 6), which is elevated in infection and flogosis [16, 19]; (ii) increasing cochlear blood flow [20] thereby avoiding cochlear ischemia [21]; (iii) avoiding noise-induced hearing loss [22]; (iv) regulating protein synthesis in the inner ear [23]. There the vascular stria regulates Na/K secretion in order to maintain endocochlear potential; it is the most frequent site of injury in the ISSHL [24]. Systemic steroid therapy improves vascular stria function and may preserve its morphology and therefore its potential for recovering from ISSHL [25].

The first report of IST in the treatment of ISSHL was by Silverstein in 1996 [26] followed by Parnes in 1999 [27]. Several other reports have been published since this initial report, the majority form 2001 [9, 10, 12, 13, 28–37]. It is demonstrated that intratympanic infusion of steroids leads to a much higher perilymphatic concentration, as compared to the systemic route. Moreover, a substantial basal-apical concentration gradient of steroid in the scala tympani perilymph has been found after round window application [38, 39].

Usually, intratympanic steroids are used in three main protocols, as initial treatment, as adjunctive treatment given concomitantly with systemic steroids, and as salvage treatment after failure of standard therapy.

The different criteria of hearing improvement and the wide variability of treatment protocols hinder the interpretation of the results. However, according to both randomized [10, 28–30, 34] and nonrandomized trials [31–33], IST, as first-line therapy, seems to be a valuable solution in refractory ISSHL, at least as effective as systemic steroids. According to our study, in literature some studies report that IST appear to be more effective in the hearing loss on the low frequencies [31, 35, 36]. Since the intratympanic steroid spreads into the perilymph through the round window, it would be actually expected that hearing improvement might occur in high frequencies (basal turn of the cochlea) than in low frequencies (apex of the cochlea). The differential vulnerability of basal and apical hair cells seems to explain this clinical result. The basal turn of the cochlea is more vulnerable to trauma and free radicals than the apical turn; in daily clinical practice, the hearing loss from noise, ototoxic drugs, or trauma easily occurs in the high frequencial range involving the cochlear base. Besides, the outer and inner hair cells of the cochlear base develop ultrastructural anomalies more quickly than those in the apical turns following severe or total cochlear ischemia [40, 41].

Although the reports about the combination of topical and systemic therapy are controversial [10, 31], the last review of the literature confirm that IST can be a reasonable alternative for patients who cannot tolerate systemic therapy or in the failure of intravenous treatment [37].

## 5. Conclusions

Difficulty in proving safety and efficacy of a single modality of IST is present in all studies on ISSH, due to a multiple treatment protocols, a variable rate of recovery, and a high rate of spontaneous recovery. Moreover, the hearing losses less than 90 dB, the involvement of the low frequencies, and the earlier IST seem to influence positively the hearing recovery, although the success could be attributed to the natural history of the disease.

More well-controlled clinical trials and standard criteria of hearing recovery are required to document the real efficacy of this option in the treatment of ISSHL and to determine the most appropriate use and the correct timing and dosage of this therapeutic modality in the emerging field of inner ear medicine delivery.
